# Exploring the potential causal relationship between lipid metabolism and oral carcinoma in situ: A 2-sample Mendelian randomization study

**DOI:** 10.1097/MD.0000000000049851

**Published:** 2026-07-24

**Authors:** Haifeng Zhou, Zongjun Ma, Huimin Lei, Yuqi Zhou, Hongguang Zhu

**Affiliations:** a Department of Stomatology, Weifang People’s Hospital, Weifang, Shandong, China.

**Keywords:** lipid metabolism markers, Mendelian randomization analysis, oral carcinoma in situ

## Abstract

Oral carcinoma in situ (OCIS) is a Stage 0 precancerous condition with a significant risk of developing into invasive oral squamous cell carcinoma. Despite this, the metabolic processes driving its onset and progression remain largely unknown. Lipid metabolism, a crucial factor in the development and behavior of various cancers, has been extensively studied. However, its exact role in OCIS has not been clearly defined, highlighting the need for further research in this area. The present study is designed to investigate the potential causal associations between specific lipid metabolic markers and OCIS through the application of Mendelian randomization (MR) analysis. Our statistical analyses indicated nominal positive correlations between 2 lipid metabolic markers and the risk of OCIS. Specifically, polyunsaturated fatty acids and total cholesterol levels in very large high-density lipoprotein were associated with increased OCIS risk, with odds ratios of 2.32 (nominal *P* value = .0463) and 2.43 (nominal *P* value = .0439) per 1 standard deviation in this marker, respectively. Conversely, the phospholipid-to-total lipid ratio in small very low-density lipoprotein showed a nominal inverse association with OCIS risk, with an odds ratio of 0.31 (nominal *P* value = .0408) per 1 standard deviation in this marker. False discovery rate correction was applied for multiple testing, and no associations reached study-wide significance. Subsequent sensitivity analyses confirmed the robustness of these nominal findings, and no substantial evidence of horizontal pleiotropy was detected for 3 of the lipid metabolic marker-OCIS associations. This exploratory study identifies 3 lipid metabolic markers (polyunsaturated fatty acids, total cholesterol levels in very large high-density lipoprotein, the phospholipid-to-total lipid ratio in small very low-density lipoprotein) that are consistent with a possible nominal effect on OCIS risk.

Summary-level genome-wide association study data were used for exposures (233 lipid metabolic markers, 136,016 European-ancestry participants, genome-wide association study Catalog accession numbers GCST90301941-GCST90302173) and outcome (OCIS, 71 cases and 378,725 controls of European-ancestry participants from the FinnGen project, release version R12, data accessed in November 2024). Instrumental variables were selected based on genome-wide significance (*P* < 5 × 10^−8^), linkage disequilibrium (*R*^2^ < 0.001, 10,000 kb window), and *F*-statistic > 10. The primary MR analysis used the inverse-variance weighted method. Sensitivity analyses included MR-Egger regression, weighted median, MR-Pleiotropy RESidual Sum and Outlier, and leave-one-out tests.

## 1. Introduction:

Oral carcinoma in situ (OCIS) is a Stage 0 preinvasive malignant lesion with a tendency to progress into invasive oral squamous cell carcinoma (OSCC). OSCC encompasses a diverse group of cancers affecting various anatomical regions of the oral cavity, such as the lips, buccal mucosa, gingiva, alveolar ridge, floor of the mouth, anterior tongue, retromolar trigone, and hard palate.^[1–3]^ The prevalence of OSCC has increased in the United States.^[4]^ However, conventional treatments are often associated with significant adverse effects and may not be effective for some patients.^[5,6]^ Epidemiological studies have linked OCIS to risk factors such as tobacco use, alcohol consumption, and betel nut chewing in specific regions. It is a rare, noninvasive condition with a variable natural history.^[7]^ The management of OCIS through biopsy remains a topic of ongoing debate among clinical professionals.

In recent years, the potential regulatory role of lipid metabolism in cancer development and progression has garnered significant scholarly attention.^[8]^ Studies have provided evidence that dysregulated lipid metabolism can promote tumor cell proliferation and metastasis by affecting critical biological processes, including cell membrane composition, intracellular signaling pathways, and cellular energy balance.^[9]^ For example, high triglyceride levels have been linked to an increased risk of various cancers.^[10]^ However, most existing research on the interaction between lipid metabolism and cancer has focused on well-studied tumor types, such as breast cancer, non-small cell lung cancer, and prostate cancer.^[11]^ The specific relationship between lipid metabolic disturbances and OCIS remains largely unexplored and poorly understood.

Mendelian randomization (MR) is a powerful analytical tool used to investigate potential causal relationships between modifiable exposures or risk factors and clinical outcomes. Compared to conventional epidemiological methods, MR has a unique advantage in reducing confounding biases that can compromise the validity of causal inferences in traditional studies. As a result, MR has become increasingly popular and reliable for evaluating and identifying plausible causal associations.^[12]^ To date, MR has provided valuable mechanistic and epidemiological insights into the causal roles of various exposures and risk factors across a wide range of human diseases.

Despite its widespread use in cardiovascular and metabolic disorder research, the application of MR in exploring the causal links between lipid metabolism and specific cancers,including OCIS, is relatively limited. This study aimed to test whether genetically predicted levels of 233 circulating lipid metabolic markers are causally linked to OCIS risk using 2-sample MR analysis. We hypothesized that specific lipid metabolic traits may be associated with OCIS susceptibility, providing novel insights for biomarker development and mechanistic research.

## 2. Materials and methods

### 2.1. Research design

**Figure 1.**
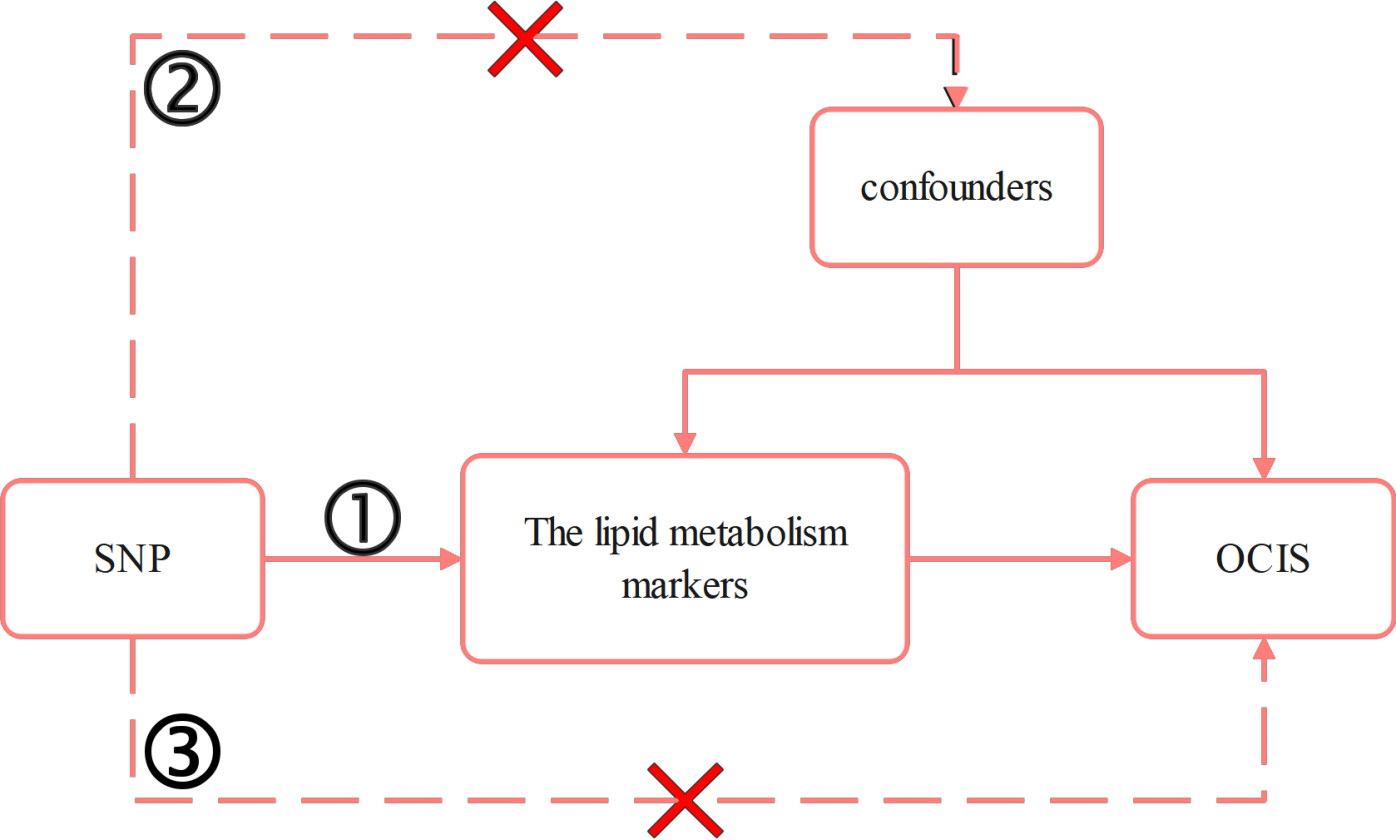
The 3 key assumptions of MR research are: the SNP is closely associated with specific lipid metabolism; the SNP is independent of other known confounding factors; and the SNP influences the risk of OCIS only through lipid metabolism. The symbol X indicates that the SNP chosen as an instrumental variable is not directly related to confounding factors or outcomes. MR = Mendelian randomization, OCIS = oral carcinoma in situ, SNP = single nucleotide polymorphism.

The application of MR methodologies relies on 3 core assumptions: the association assumption,the independence assumption,and the exclusion-restriction assumption (Fig. 1).^[13]^ These assumptions are defined as follows: the chosen instrumental variables (IVs) must have a statistically significant association with the exposure of interest; the IVs should be genetically independent of any confounding factors that could influence the exposure-outcome relationship; the IVs should affect the outcome variable only through their impact on the exposure, with no direct or indirect effects independent of the exposure. In this study, these fundamental assumptions were rigorously adhered to throughout the 2-sample MR analysis to ensure the validity of the selected IVs and the robustness of the primary findings. The study was exploratory, with the primary analysis using the inverse-variance weighted (IVW) method, while sensitivity analyses included MR-Egger (horizontal pleiotropy detection), weighted median (robust to weak IVs), MR-Pleiotropy RESidual Sum and Outlier (outlier detection), and leave-one-out (assessing IV influence).

**Figure 2.**
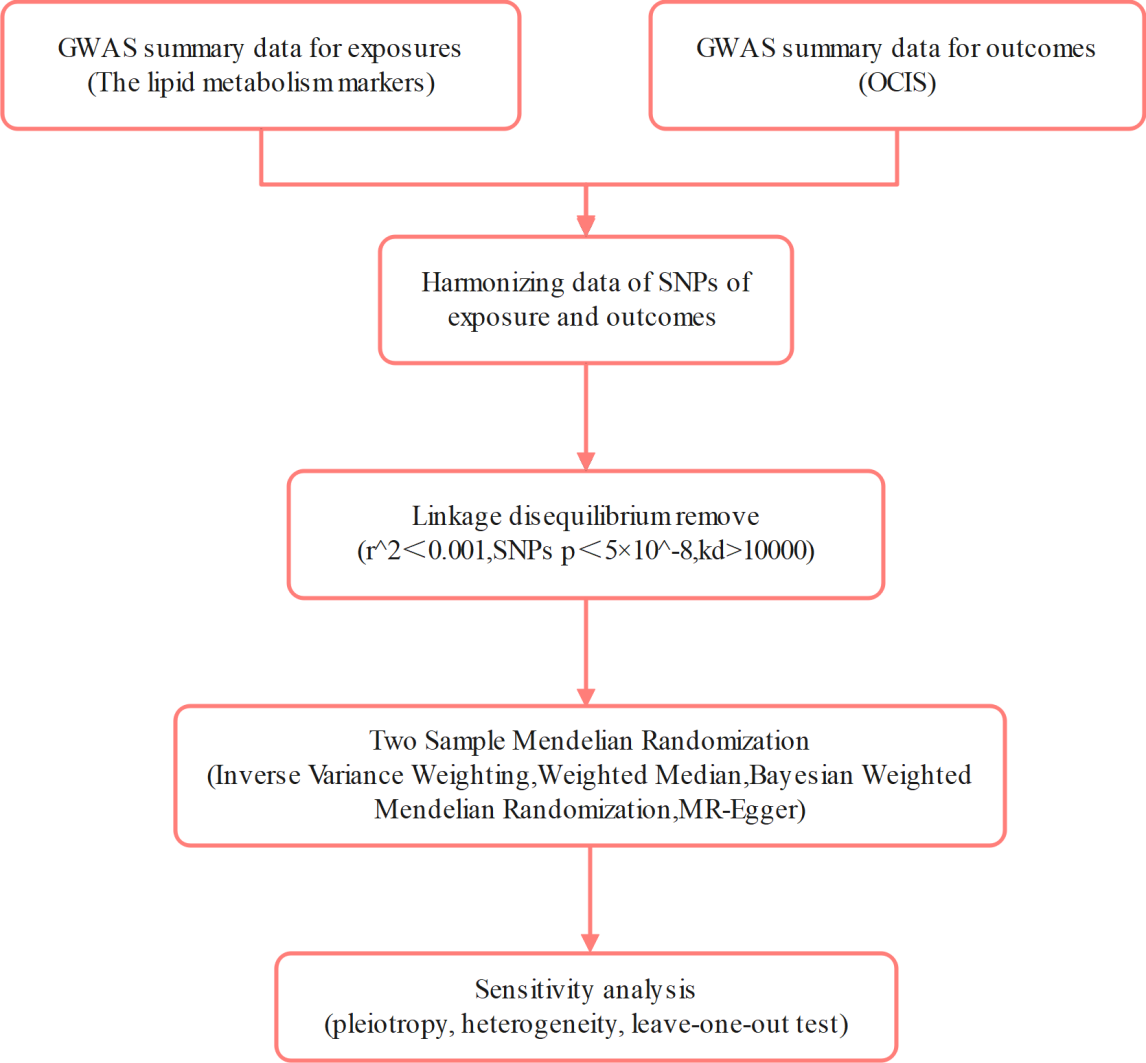
Workflow of the study. GWAS = genome-wide association study, MR = Mendelian randomization, OCIS = oral carcinoma in situ, SNP = single nucleotide polymorphism.

In this study, we conducted a 2-sample MR analysis using a panel of 233 circulating metabolic markers. Summary-level data from genome-wide association studies (GWAS) were used to evaluate potential causal associations between these markers and OCIS, with the aim of identifying metabolic traits that have statistically nominal causal links with OCIS. Additionally, we performed sensitivity analyses to assess the robustness and reliability of the primary MR results. The overall workflow of this investigation is schematically illustrated in Figure 2.

### 2.2. Data sources

Exposure data: For the GWAS-derived data on lipid metabolism, a large-scale study with 136,016 participants was selected. The univariate summary statistics from this study (released in March 2024) are publicly accessible in the GWAS Catalog (accession numbers GCST90301941 -GCST90302173). The study systematically analyzed 233 circulating metabolic markers using nuclear magnetic resonance spectroscopy, a highly precise technique for metabolic profiling.^[14]^

Outcome data: The OCIS-related genetic data used in this study were obtained from the FinnGen project (release version R12, data accessed in November 2024), which includes 378,796 European-ancestry participants (71 cases and 378,725 controls). The FinnGen study is a large-scale,ongoing biobank-based initiative that integrates germline genotype data from Finnish biobanks with comprehensive health record data from Finnish national health registries.^[15]^ All datasets used in this study are publicly available via the FinnGen documentation website (https://finngen.gitbook.io/documentation/) and have undergone ethical review and approval from the respective institutional ethics committees. As per standard research protocols for secondary analyses of preapproved, publicly accessible data, no additional ethical clearance was required for this study.

Sample overlap and reference panel: No sample overlap between exposure and outcome datasets was reported by the original studies. The 1000 Genomes Project (Phase 3, European ancestry) was used as the reference panel for linkage disequilibrium (LD) clumping and allele frequency estimation during IVs single nucleotide polymorphism (SNP) selection.

### 2.3. Selection and validation of IV SNPs

The selection process of SNPs associated with 233 circulating metabolic markers in this study was rigorous and methodical, guided by 3 key criteria. Firstly, SNPs had to meet the stringent threshold for genome-wide significance, with a *P* value below 5 × 10^−^8, ensuring high statistical significance. Additionally, the genetic independence of the chosen SNPs was carefully assessed through LD analysis, setting a threshold at *R*^2^ < 0.001 to exclude redundant genetic signals. Any SNPs showing LD with others within a 10000 kb genomic window and having higher *P* values than the selected SNPs were excluded to maintain the integrity of the selection process. Moreover, to prevent weak instrument bias, a crucial confounding factor in MR analyses, the strength of the IVs was evaluated using the *F*-statistic. IVs with an *F*-statistic value exceeding 10 were deemed suitable for analysis, confirming the absence of weak instrument bias. This step further ensured the validity of the MR association assumption, crucial for accurate interpretation of the results. The calculation of the *F*-statistic involved a formula that considered factors such as sample size, the number of IVs, and the proportion of variance explained by the candidate IVs. Overall, the meticulous selection process implemented in this study guarantees the robustness and reliability of the findings related to metabolic markers and their genetic associations.

Table S1–S3, Supplemental Digital Content 1 lists details of IVs for the 3 focal markers: SNPs, effect alleles, beta coefficients, standard error of the mean, and *F*-statistics.

### 2.4. Two-sample MR analysis

This study utilized a 2-sample MR analysis to investigate the potential causal links between specific lipid metabolic markers and OCIS. Various MR analytical approaches were employed, such as the random-effects IVW method, the weighted median method, and the MR-Egger regression method. IVW was prespecified as the primary estimator. Genome build was standardized to hg19. In the data preparation stage, the harmonize function from the TwoSampleMR package was used to remove palindromic sequences. The study quantified the extent of causal effects and presented them as odds ratios (OR) per 1 standard deviation (SD). Results from this analysis shed light on the potential connections between lipid metabolism and OCIS.

The conventional random-effects IVW method frequently used in MR analyses can be influenced by biases due to invalid IVs or horizontal pleiotropy, potentially undermining the reliability of causal conclusions. To mitigate this concern and uphold the validity of primary results obtained through IVW, supplementary sensitivity analyses were carried out. These additional assessments aim to enhance the robustness and accuracy of the research findings, ensuring a more dependable causal inference.

The sensitivity analyses conducted in the present study encompassed 3 key complementary steps, namely the assessment of horizontal pleiotropy, heterogeneity testing, and a “leave-one-out” sensitivity approach: all designed to rigorously validate the reliability of the primary MR findings.

First, to detect the presence of horizontal pleiotropy, MR-Egger regression was employed. A statistical threshold of *P* value < .05 was adopted to denote the presence of nominal horizontal pleiotropy between the exposure and the outcome.

Second, Cochran *Q* test was utilized to evaluate heterogeneity among the selected IVs. A *P* value > .05 was interpreted as indicating the absence of statistically nominal heterogeneity across the IVs, thereby suggesting that heterogeneity does not introduce meaningful bias to the study’s causal inference.

A “leave-one-out” sensitivity analysis was conducted to investigate how individual IVs affected the overall causal effect estimates on the relationship between lipid metabolism and OCIS. Each SNP acting as an IV was systematically removed 1 at a time,and the causal effect of the remaining SNPs on the association was recalculated. This process allowed for an assessment of the consistency of the primary causal effect estimates even after excluding a single SNP, ensuring the reliability of the study’s results. The analysis aimed to verify the stability of the findings by examining the impact of each IV on the overall causal effect estimates.

All statistical analyses involved in the present study were implemented using the “TwoSampleMR” package, a specialized tool for MR analyses, within the R software environment (version 4.3.2; R Foundation for Statistical Computing).

## 3. Results

The study explored potential causal relationships between circulating metabolic markers and the occurrence of OCIS. Through a comprehensive 2-sample MR analysis of 233 metabolic markers, 3 specific lipid markers stood out for their potential impact on OCIS. These lipid markers include polyunsaturated fatty acids (GCST90302071), total cholesterol levels in very large high-density lipoprotein (HDL) (GCST90302126), and the phospholipid-to-total lipid ratio in small very low-density lipoprotein (VLDL) (GCST90302113).

The study showed that polyunsaturated fatty acids (PUFAs) were nominally positively associated with the risk of OCIS, with an OR of 2.32 per 1 SD and a nominal *P* value of .0463. Furthermore, total cholesterol levels in very large HDL were also linked to the risk of OCIS, with an OR of 2.43 per 1 SD in this marker and a nominal *P* value of .0439. On the other hand, the ratio of phospholipids to total lipids in VLDL was nominally inversely correlated with the risk of OCIS, with an OR of 0.31 per 1 SD in this marker and a nominal *P* value of .0408. All nominal *P* values in the present study were corrected using the false discovery rate method for multiple testing, and none of the observed associations achieved study-wide statistical significance after correction. All numerical data are summarized in Table 1.

**Table 1:** Associations between the lipid metabolism markers and the risks of OCIS.

Risk factors		MR-Egger	Weighted median	IVW
Polyunsaturated fatty acids	OR (95% CI)	3.36 (0.80–14.15)	3.37 (0.94–12.08)	2.32 (1.01–5.30)
	*P* value	.1040	.0622	.0463
Total cholesterol levels in very large HDL	OR (95% CI)	2.55 (0.58–11.27)	2.81 (0.78–10.12)	2.43 (1.02–5.75)
	*P* value	.2220	.1150	.0439
Phospholipids to total lipids ratio in small VLDL	OR (95% CI)	0.59 (0.07–5.08)	0.67 (0.14–3.19)	0.32 (0.10–0.98)
	*P* value	0.6360	.6150	.04532

CI = confidence interval, HDL = high-density lipoprotein, IVW = inverse-variance weighted, MR = Mendelian randomization, OCIS = oral carcinoma in situ, OR = odds ratio, VLDL = very low-density lipoprotein. All *P* values presented in this table are nominal *P* values.

**Figure 3.**
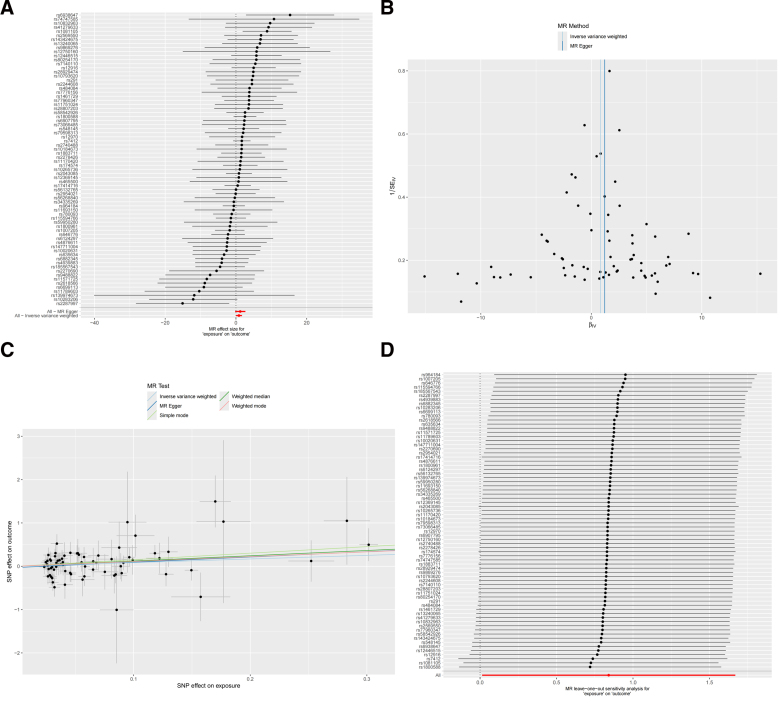
The causal effect of PUFAs levels on the risk of OCIS. (A) Forest plot, (B) funnel plot, (C) scatter plot, (D) and leave-one-out sensitivity analysis of the causal effect of PUFAs levels on the risk of OCIS. MR = Mendelian randomization, OCIS = oral carcinoma in situ, PUFA = polyunsaturated fatty acids, SE = standard error, SNP = single nucleotide polymorphism.

**Figure 4.**
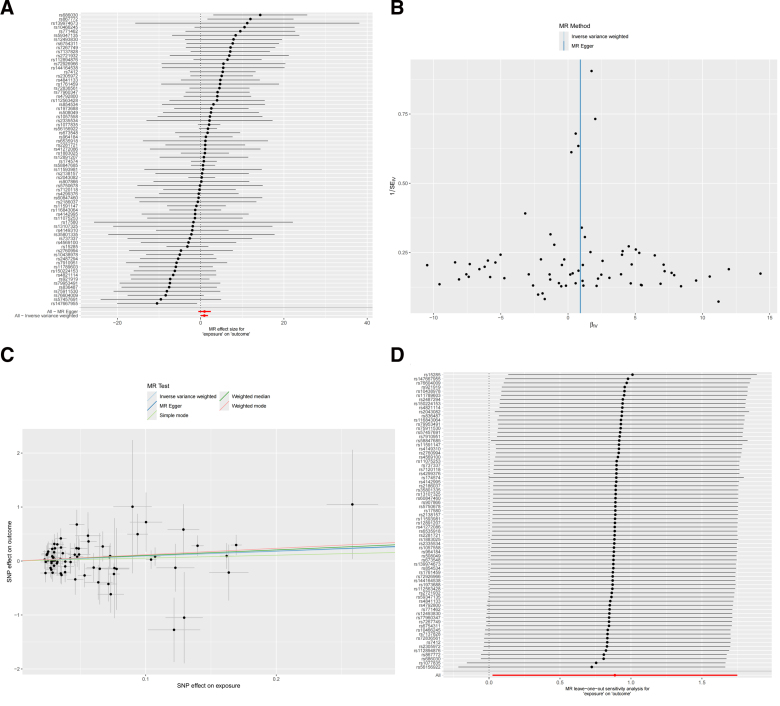
The causal effect of total cholesterol levels in very large HDL on the risk of OCIS. (A) Forest plot, (B) funnel plot, (C) scatter plot, (D) and leave-one-out sensitivity analysis of the causal effect of total cholesterol levels in very large HDL on the risk of OCIS. HDL = high-density lipoprotein, MR = Mendelian randomization, OCIS = oral carcinoma in situ, SE = standard error, SNP = single nucleotide polymorphism.

**Figure 5.**
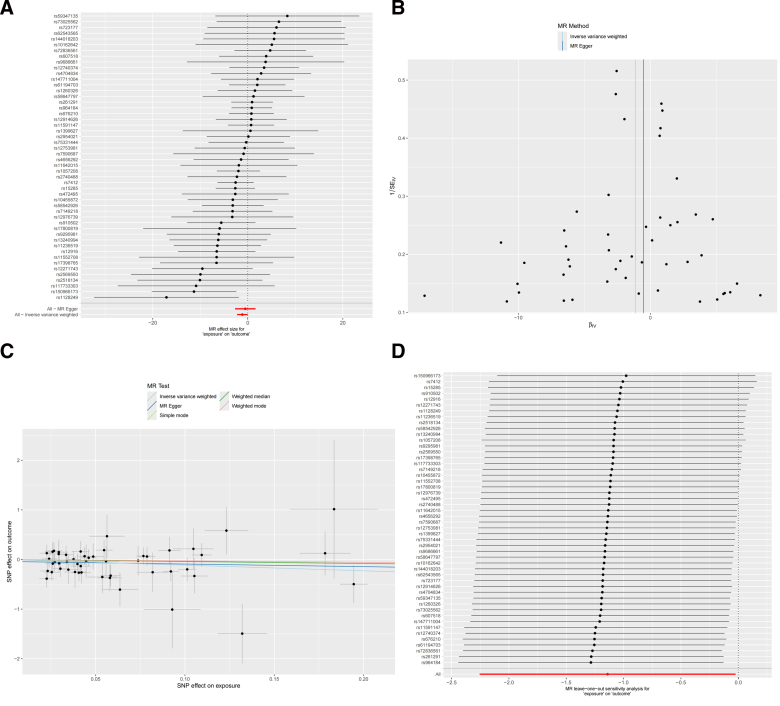
The causal effect of the ratio of phospholipids to total lipids in small VLDL on the risk of OCIS. (A) Forest plot, (B) funnel plot, (C) scatter plot, and (D) leave-one-out sensitivity analysis of the causal effect of the ratio of phospholipids to total lipids in small VLDL on the risk of OCIS. MR =Mendelian randomization, OCIS = oral carcinoma in situ, SE = standard error, SNP = single nucleotide polymorphism, VLDL = very low-density lipoprotein.

A thorough investigation was carried out to confirm the strength and dependability of the causal relationship findings mentioned earlier. Various sensitivity analyses were performed, focusing on the 3 specific lipid metabolic markers that were identified. The outcomes of these rigorous analyses are outlined in Tables 2 and 3. Furthermore, to provide a visual representation and authentication of the study’s analytical results, supplementary visualization materials such as forest plots, funnel plots, scatter plots, and leave-one-out sensitivity analysis plots can be found in Figures 3 to 5.

**Table 2:** Pleiotropy test results for selected SNPs.

Risk factors	Egger intercept	Pleiotropy test beta (SE)	Pleiotropy test *P* value
Polyunsaturated fatty acids	−0.028	0.0452	.540
Total cholesterol levels in very large HDL	−0.003	0.0437	.937
Phospholipids to total lipids ratio in small VLDL	−0.040	0.0600	.511

HDL = high-density lipoprotein, SE = standard error, SNPs = single nucleotide polymorphisms, VLDL = very low-density lipoprotein.

**Table 3:** Heterogeneity test results for selected SNPs.

Risk factors	Heterogeneity (MR-Egger)		Heterogeneity (IVW)	
	Cochran *Q* (df)	Heterogeneity test *P*-value	Cochran *Q* (df)	Heterogeneity test *P* value
Polyunsaturated fatty acids	66.606 (70)	.592	66.985 (71)	.613
Total cholesterol levels in very large HDL	69.510 (70)	.494	69.516 (71)	.527
Phospholipids to total lipids ratio in small VLDL	42.901 (48)	.681	43.338 (49)	.701

HDL = high-density lipoprotein, IVW = inverse-variance weighted, MR = Mendelian randomization, SNPs = single nucleotide polymorphisms, VLDL = very low-density lipoprotein.

The MR-Egger regression analysis results indicated that the 3 lipid metabolic markers under study did not show nominal horizontal pleiotropy, as shown in Table 2. This suggests that the IVs chosen for these markers are reliable and not biased by other factors. Therefore, the conclusions drawn about the nominal relationship between these markers and the risk of OCIS are trustworthy.

The results of Cochran *Q* test revealed that there was no nominal heterogeneity among SNPs as shown in Tables 3. Overall, the study indicates a stable nominal association between the lipid metabolic markers and OCIS risk.

The MR-Pleiotropy RESidual Sum and Outlier test was used to analyze 3 lipid metabolic markers: PUFAs, total cholesterol in very large HDL, and the ratio of phospholipids to total lipids in small VLDL. The results revealed that none of these markers exhibited statistically nominal pleiotropy, with nominal *P* values of .65, .731, and .52, respectively. Additionally, no nominal outlier SNPs were identified among the IVs corresponding to these markers, indicating their robustness and reliability for analyzing these nominal associations..

The leave-one-out sensitivity analysis revealed that each genetic variant has a consistent impact on the causal effect estimates, indicating the stability and credibility of these nominal study results.

## 4. Discussion

The current study utilized a 2-sample MR analysis to shed light on the potential causal relationships between 3 important lipid markers and the risk of OCIS. Specifically, it was found that PUFAs and total cholesterol levels in very large HDL were nominally positively linked to an increased risk of OCIS, while the ratio of phospholipids to total lipids in small VLDL showed a nominal inverse relationship with the risk of OCIS. However, this study’s findings are exploratory, and after false discovery rate (FDR) correction, no signals meet study-wide significance.

The analysis conducted in IVW revealed a noteworthy correlation between PUFAs and the risk of OCIS. The results indicated a statistically nominal positive causal relationship,with an OR of 2.32 per 1 SD in this marker and a corresponding nominal *P* value of .0463. PUFAs are recognized for their pivotal roles in regulating various cancer-related processes, such as cell proliferation, apoptosis, migration, invasion, metabolic reprogramming, epigenetic modifications, and modulation of the tumor immune microenvironment. Past research has demonstrated that PUFAs impact cancer progression through diverse mechanisms, including altering cell membrane lipid composition, engaging with lipid-sensitive receptors on cell membranes,translocating into the cell nucleus to influence cancer-related gene expression,and contributing to the synthesis and metabolism of bioactive lipid mediators.^[16]^

The IVW analysis indicates a notable positive causal relationship between high levels of total cholesterol in very large HDL and an increased risk of OCIS, with an OR of 2.43 (nominal *P* value = .0439) per 1 SD increase in this marker. Previous studies have shown that dysregulation of cholesterol metabolism can affect various stages of tumor progression, such as tumor formation, cancer cell growth, and spread to other parts of the body. Cholesterol’s impact on important cellular and molecular processes, including the regulation of immune response against tumors, control of cell death, maintenance of cancer stem cells, and DNA damage response, all contribute to its regulatory role in cancer development.^[17]^

The IVW analysis uncovered an essential discovery indicating a noteworthy nominal inverse causal link between the phospholipids to total lipids ratio in small VLDL and the risk of OCIS. This defensive mechanism could operate by influencing crucial biological mechanisms associated with the development of tumors, such as regulating lipid distribution in bodily tissues or transforming cellular metabolic routes that promote the growth and persistence of malignant cells.

The biological plausibility of the observed associations supports further investigation, but the findings do not justify claims of therapeutic or preventive utility at this stage. Future research is needed to validate these signals before any clinical applications can be considered.

Dysregulation of VLDL metabolism, which includes issues in its production, release, lipid content, and breakdown, has been associated with the development of various human diseases like heart problems and different types of cancer.^[18]^ This highlights the importance of understanding and addressing VLDL metabolism abnormalities in preventing and managing diseases effectively, particularly cancer,by targeting the inflammatory and oxidative stress pathways influenced by VLDL particles.^[19]^

Notably, epidemiological and proteomic studies have documented that the expression or posttranslational modification of numerous proteins that are constitutively associated with HDL particles is significantly altered in patients with various malignancies. This observation implies a potential link between dysregulated HDL particle composition and cancer pathogenesis.^[20]^

Despite the promising and novel insights derived from the present study, several inherent limitations must be rigorously acknowledged when interpreting its findings.

The credibility of causal inferences in MR analyses relies heavily on meeting certain IV assumptions. These include selecting SNPs that are closely linked to the exposure, are unaffected by confounding variables, and impact the outcome solely through the exposure. Any alternative pathways, like horizontal pleiotropy, could introduce significant bias and jeopardize the accuracy of the causal estimates. Therefore, ensuring the fulfillment of these core IV assumptions is essential for reliable inference in MR analyses.

Genetic diversity among different ancestral populations can influence how strongly or in what way specific genetic variations are associated with certain traits. This study mainly focuses on data from individuals of European descent, making it unclear how applicable the findings are to non-European ethnicities or populations with diverse genetic backgrounds. This limitation hinders the broader applicability of the results and raises questions about how well the observed associations can be generalized to other populations.

Third, conventional MR analyses typically operate under the assumption of a linear dose-response relationship between the exposure and the outcome. If the true underlying relationship is nonlinear, this linearity assumption may lead to misestimation of causal effects and potentially erroneous conclusions regarding the nature of the association.

The quality and strength of MR results rely heavily on the accuracy and completeness of the underlying GWAS data. Limitations in the original GWAS, like residual confounding or measurement errors, can affect the MR analysis and lead to biased outcomes. It is crucial to ensure the integrity of the GWAS data to obtain reliable and robust MR results, ultimately enhancing the validity of the study findings.

While MR analysis allows for the formation of hypotheses about causal relationships, it is a correlational-epidemiological method and does not directly reveal the precise molecular or cellular mechanisms behind lipid metabolism-OCIS nominal associations. MR also does not provide information on the potential impact of targeted interventions, as it relies on genetic proxies instead of experimental manipulation. Therefore, while MR analysis is valuable for generating ideas, it has limitations in fully understanding the mechanisms and potential effects of interventions on lipid metabolism-OCIS connections.

The present study offers initial insights into the connections between lipid metabolic markers and the risk of developing OCIS. Despite these valuable findings, it is essential to acknowledge the limitations of the study. When interpreting and applying these results to broader clinical or public health settings, caution is necessary. Future research should prioritize several key areas. Firstly, efforts should focus on confirming the identified associations in more diverse ethnic groups to ensure the generalizability of the findings. Additionally, integrating these results with experimental studies can help unravel the underlying biological mechanisms connecting lipid metabolism and OCIS. Moreover, in-depth mechanistic investigations and well-designed clinical trials are essential to validate the potential use of lipid metabolic markers as targets for preventing or treating OCIS. These efforts will also help assess the safety and effectiveness of lipid-based intervention strategies. Ultimately, these comprehensive research initiatives are crucial for informing future strategies in addressing OCIS.

## 5. Conclusion

This study delves into the correlation between specific lipid metabolic markers and OCIS using a MR framework. The research uncovers noteworthy findings, demonstrating a nominal positive causal relationship between OCIS risk and PUFAs and total cholesterol levels in very large HDL. Conversely, the ratio of phospholipids to total lipids in small VLDL exhibits a nominal inverse causal association with OCIS risk. The findings provide preliminary insights for future replication studies and mechanistic investigations into lipid metabolism in OCIS.

## Author contributions

**Conceptualization:** Hongguang Zhu, Haifeng Zhou, Zongjun Ma, Huimin Lei.

**Data curation:** Hongguang Zhu, Huimin Lei, Yuqi Zhou.

**Formal analysis:** Zongjun Ma.

**Investigation:** Hongguang Zhu, Haifeng Zhou.

**Methodology:** Hongguang Zhu, Haifeng Zhou, Zongjun Ma.

**Project administration:** Hongguang Zhu.

**Resources:** Hongguang Zhu, Huimin Lei, Yuqi Zhou.

**Supervision:** Hongguang Zhu.

**Writing – review & editing:** Hongguang Zhu, Haifeng Zhou, Zongjun Ma, Huimin Lei, Yuqi Zhou.

**Writing – original draft:** Haifeng Zhou.






